# Supplementary material for: Far-East Asian Toxoplasma isolates share ancestry with North and South/Central American recombinant lineages
Source: Nat Commun. 2024 May 22;15:4278. doi: 10.1038/s41467-024-47625-6 (PMC11111807; doi:10.1038/s41467-024-47625-6)
Supplement: Supplementary file 1 — Supplementary Information [file 41467_2024_47625_MOESM1_ESM.pdf]

SUPPLEMENTARY INFORMATION

Far-East Asian *Toxoplasma* isolates share ancestry with North and South/Central American recombinant lineages.

Fumiaki Ihara[1,2,3], Hisako Kyan[4], Yasuhiro Takashima[5,6], Fumiko Ono[7], Kei Hayashi[8], Tomohide Matsuo[9], Makoto Igarashi[10], Yoshifumi Nishikawa[10], Kenji Hikosaka[11], Hirokazu Sakamoto[11], Shota Nakamura[12], Daisuke Motooka[12], Kiyoshi Yamauchi[13], Madoka Ichikawa-Seki[14], Shinya Fukumoto[10], Motoki Sasaki[15], Hiromi Ikadai[16], Kodai Kusakisako[16], Yuma Ohari[17], Ayako Yoshida[18, 19], Miwa Sasai[1,2,3], Michael E. Grigg[20], and Masahiro Yamamoto[1,2,3]

[1]Department of Immunoparasitology, Research Institute for Microbial Diseases, Osaka University, Yamadaoka, Suita, Osaka 565-0871, Japan.

[2]Laboratory of Immunoparasitology, WPI Immunology Frontier Research Center, Osaka University, Yamadaoka, Suita, Osaka 565-0871, Japan.

[3]Department of Immunoparasitology, Center for Infectious Disease Education and Research, Osaka University, Suita, Osaka 565-0871, Japan.

[4]Okinawa Prefectural Institute of Health and Environment, Uruma, Okinawa 904-2241, Japan.

[5]Faculty of Applied Biological Sciences, Gifu University, Gifu 501-1112, Japan.

[6]Center for One Medicine Translational Research, COMIT, Gifu University, Gifu 501-1112, Japan.

[7]Department of Veterinary Associated Science, Faculty of Veterinary Medicine, Okayama University of Science, Imabari, Ehime 794-8555, Japan.

[8]Laboratory of Parasitology, Faculty of Veterinary Medicine, Okayama University of Science, Imabari, Ehime 794-8555, Japan.

[9]Joint Faculty of Veterinary Medicine Kagoshima University, Kagoshima 890-0065, Japan.

[10]National Research Center for Protozoan Diseases, Obihiro University of Agriculture and Veterinary Medicine, Obihiro, Hokkaido 080-8555, Japan.

[11]Department of Infection and Host Defense, Graduate School of Medicine, Chiba University, Chiba 260-0856, Japan.

[12]Department of Infection Metagenomics, Research Institute for Microbial Diseases, Osaka University, Suita, Osaka 565-0871, Japan.

[13]Laboratory of Wildlife Management, Faculty of Agriculture, Iwate University, Morioka, Iwate 020-8550, Japan.

[14]Laboratory of Veterinary Parasitology, Faculty of Agriculture, Iwate University, Morioka, Iwate 020-8550, Japan.

[15]Laboratory of Veterinary Anatomy, Obihiro University of Agriculture and Veterinary Medicine, Obihiro, Hokkaido 080-8555, Japan.

[16] Laboratory of Veterinary Parasitology, School of Veterinary Medicine, Kitasato University, Aomori 034-8628, Japan.

[17]Division of Risk Analysis and Management, International Institute for Zoonosis Control, Hokkaido University, Sapporo, Hokkaido 001-0020, Japan.

[18]Laboratory of Veterinary Parasitic Diseases, Department of Veterinary Sciences, Faculty of Agriculture, University of Miyazaki, Miyazaki 889-2155, Japan

[19]Center for Animal Disease Control, University of Miyazaki, Miyazaki 889-2155, Japan.

[20]Molecular Parasitology Section, Laboratory of Parasitic Diseases, National Institutes of Health, National Institute of Allergy and Infectious Diseases (NIAID), Bethesda, MD, 20892, USA.

*Correspondence to myamamoto@biken.osaka-u.ac.jp)

The Supplementary Information includes:

-Supplementary Figures 1-13 and their legends

-Supplementary Tables 1-4

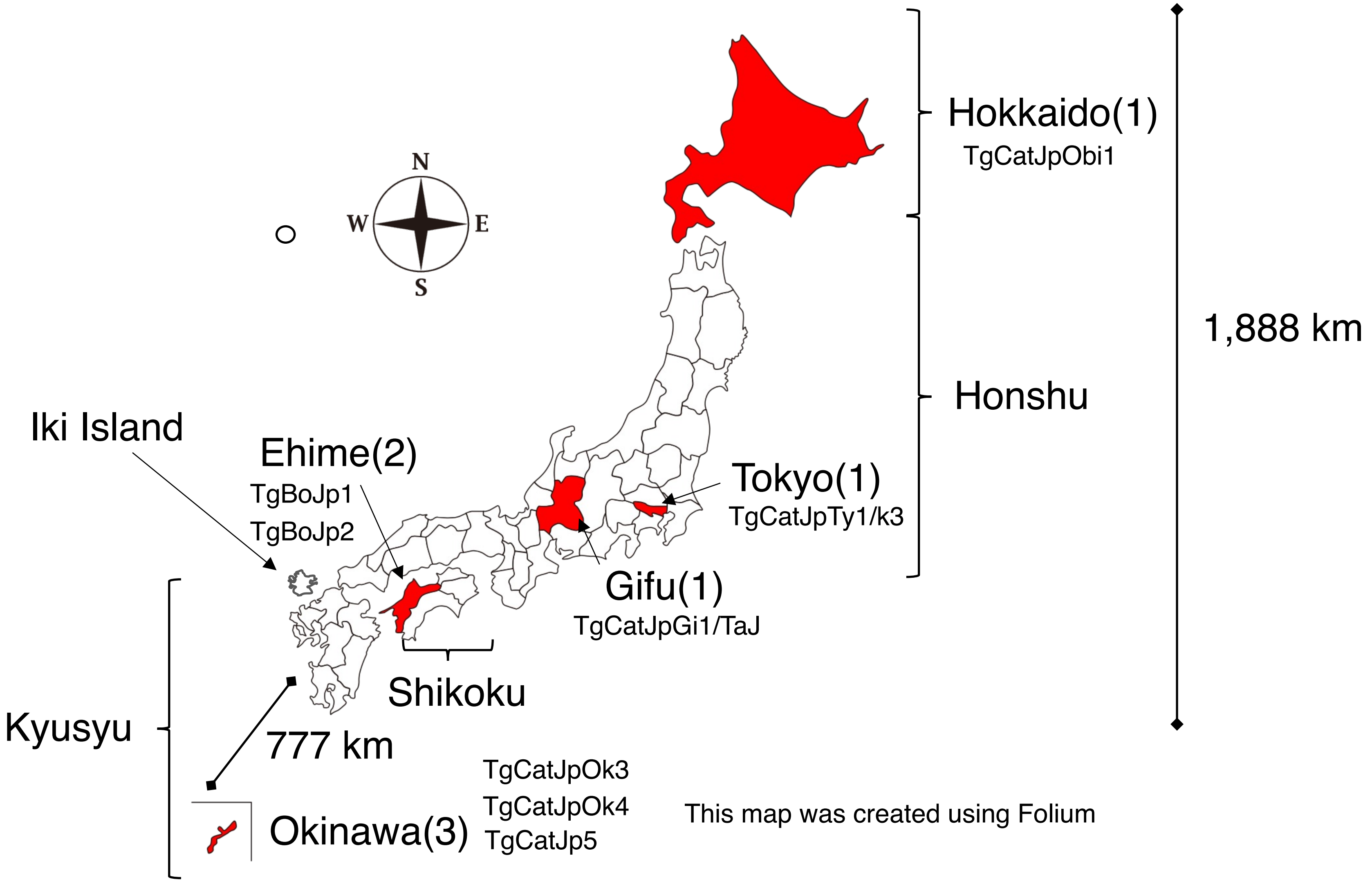

Figure S1. Geographic distribution of *T. gondii* isolates used in this study.
The locations of the Japanese strains were isolated. Japan consists of four main islands: Hokkaido, Honshu, Shikoku, and Kyushu. *T. gondii* strains were isolated from prefectures indicated by red. Black arrows indicate the name of each using Folium prefecture. The numerical values indicate the number of isolates from each location. Straight lines indicate the linear distance between the starting and ending points. This map was created created using Folium (https://github.com/python-visualization/folium)

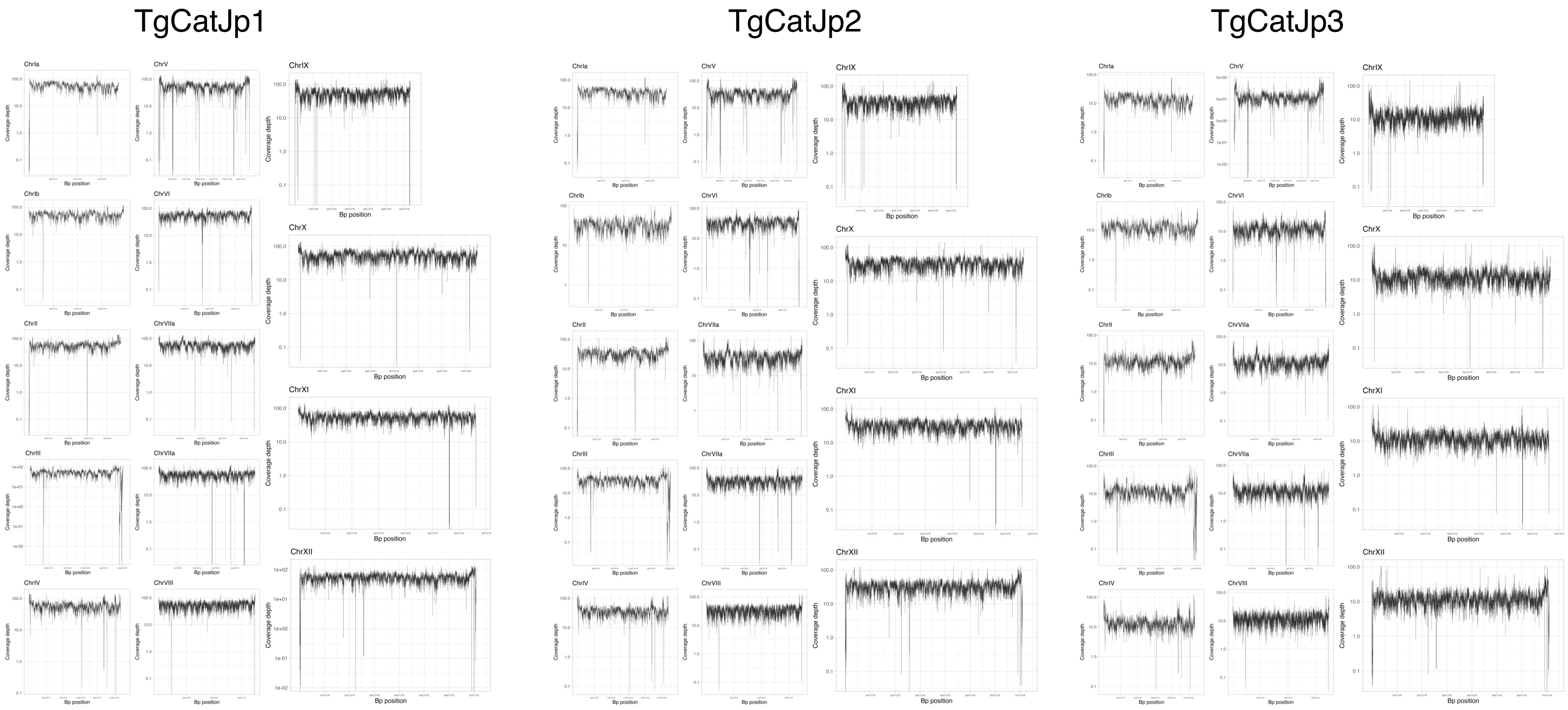
TgCatJp1
TgCatJp2
TgCatJp3
ChrIa
ChrIb
ChrII
ChrIII
ChrIV
ChrV
ChrVI
ChrVIIa
ChrVIII
ChrIX
ChrX
ChrXI
ChrXII
Coverage depth
Bp position

Continued

TgCatJp4

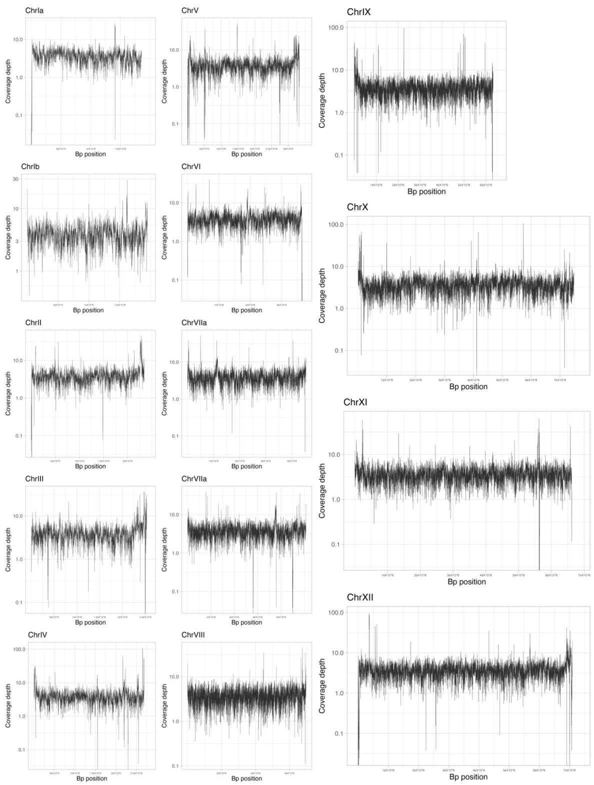

TgCatJp6

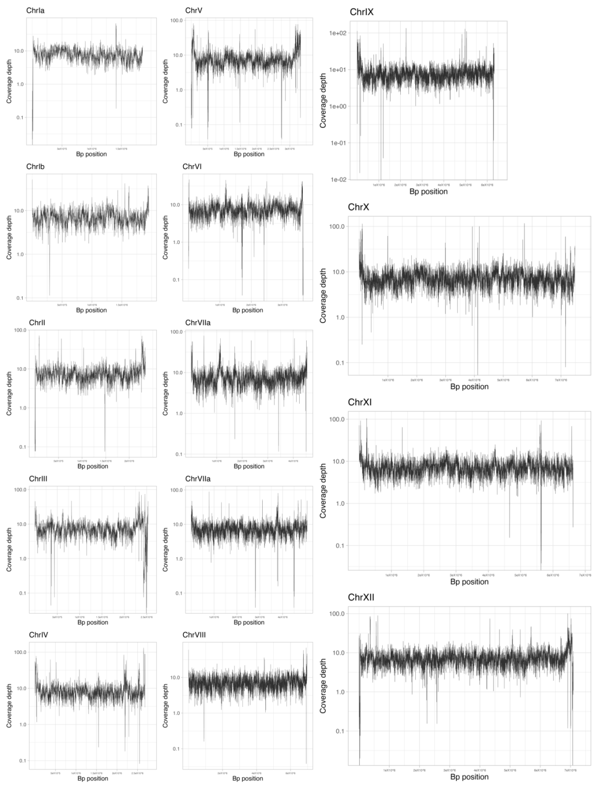

TgCatJp7

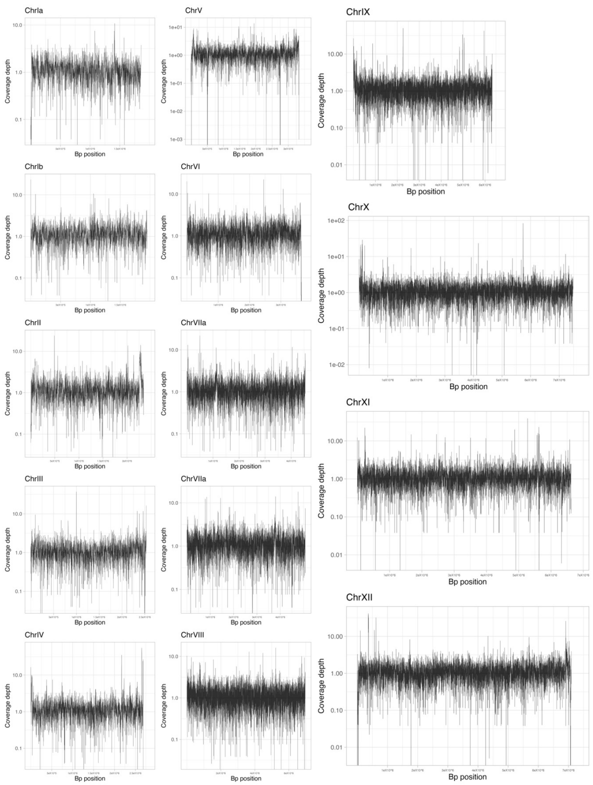

Continued

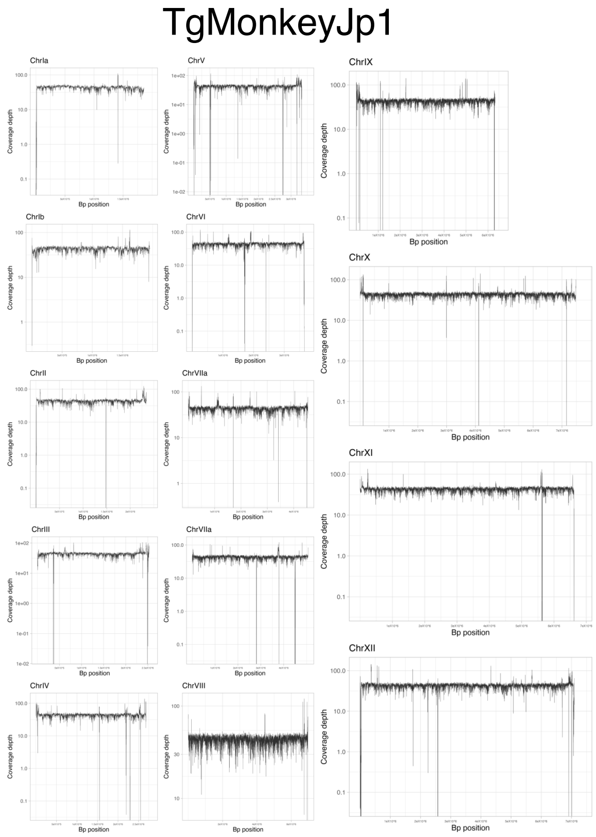

Figure S2. Coverage plot of SureSelect samples.

This set of graphs represents the sequencing depth across various chromosomes within a genome. Each panel corresponds to a different chromosome, labeled from ChrIa to ChrXII, showsing the sequencing depth at each base pair position along the chromosome.

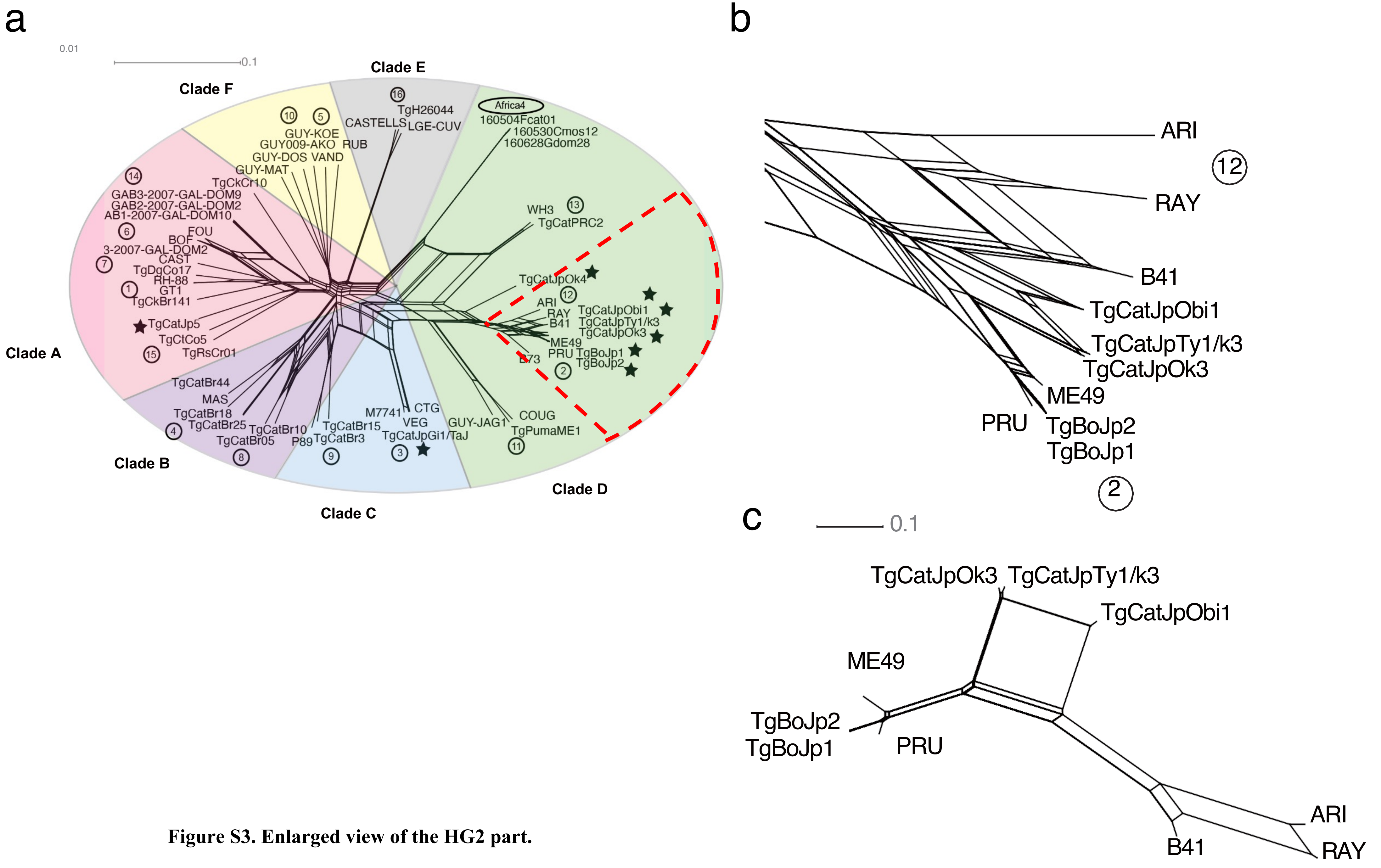

Figure S3. Enlarged view of the HG2 part.

(a) This panel shows the same Neighbor network as Fig.1a.

(b) This panel shows the magnified cutout of the dotted line from (a).

(c) Intra-group network analysis using the same isolates as (b).

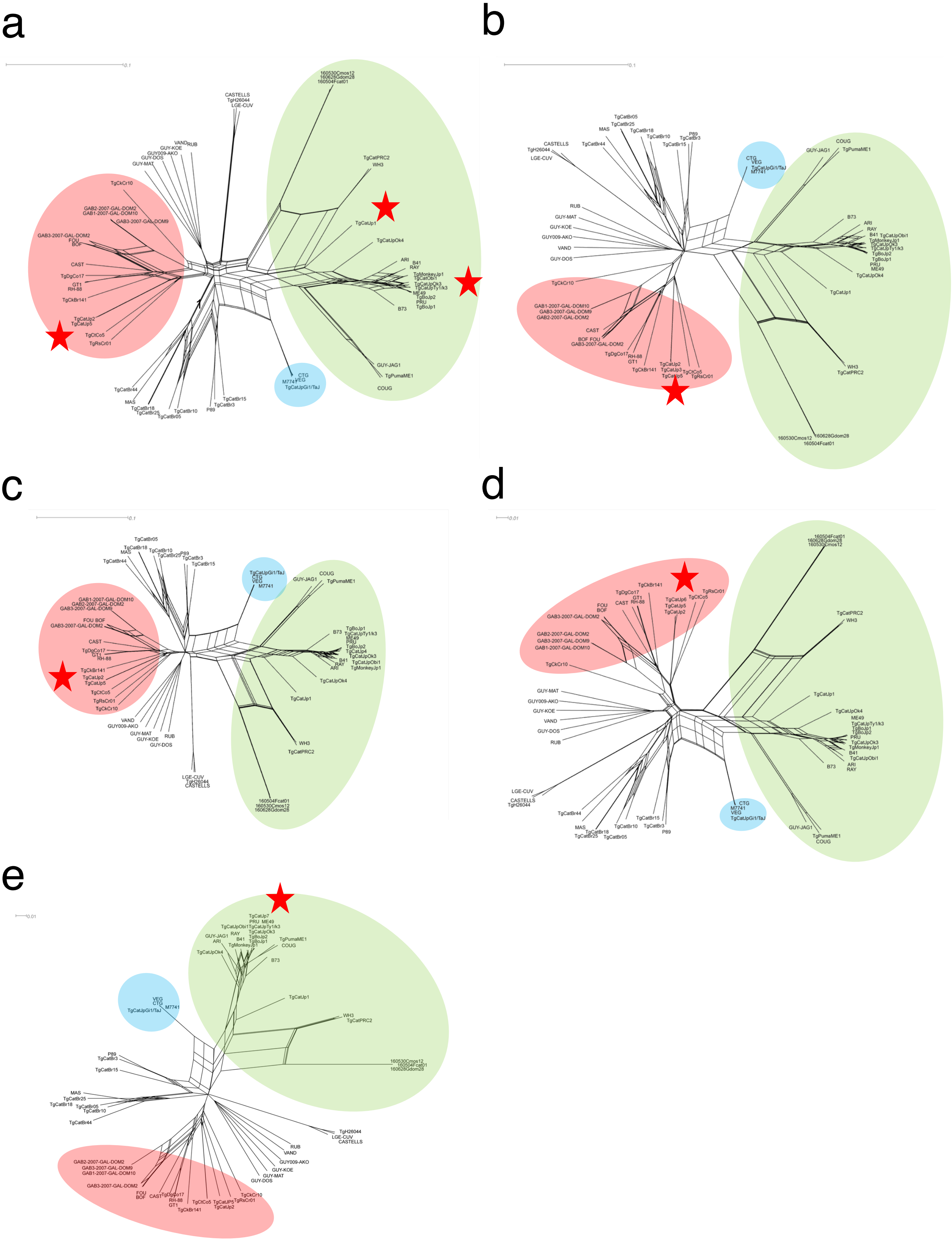

Figure S4. Phylogenetic analysis of Japanese *T. gondii* strains restored by targeted enrichment method.

Neighbor-Network with the addition of (a) TgCatJp1, TgCatJp2, TgMonkeyJP1, (b) TgCatJp3, (c) TgCatJp4, (d) TgCatJp6, (e) TgCatJp7. Asterisks indicate added Japanese strains. Color ellipses indicate major clades of *T. gondii*. Numbers indicated in each network is the number of variant sites used in the analysis.

a

Red: TgCatJp5

Blue: TgCatJpOk4

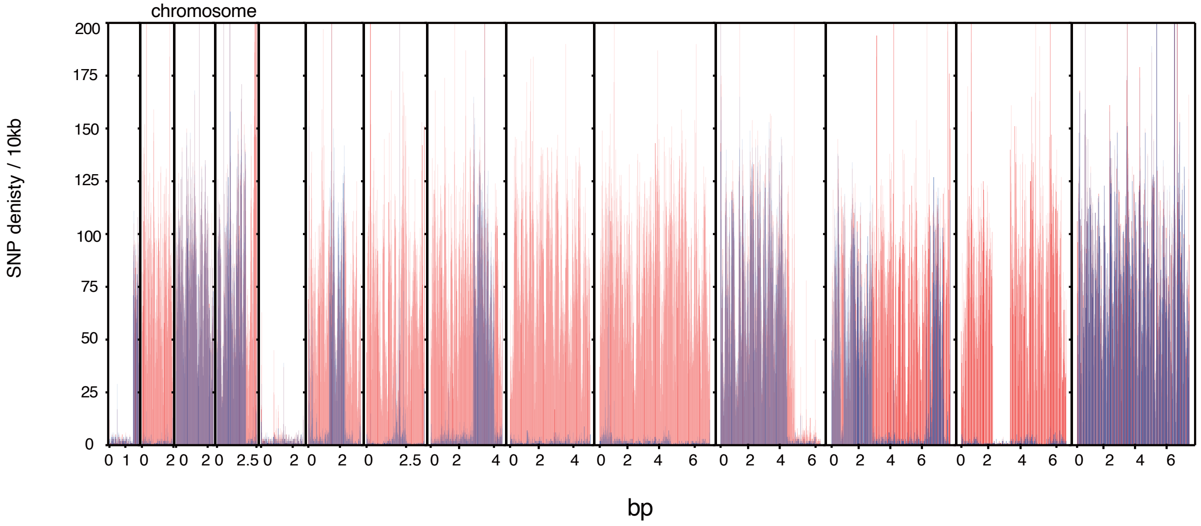

b

Red: VEG

Blue: TgCatJpGi1/TaJ

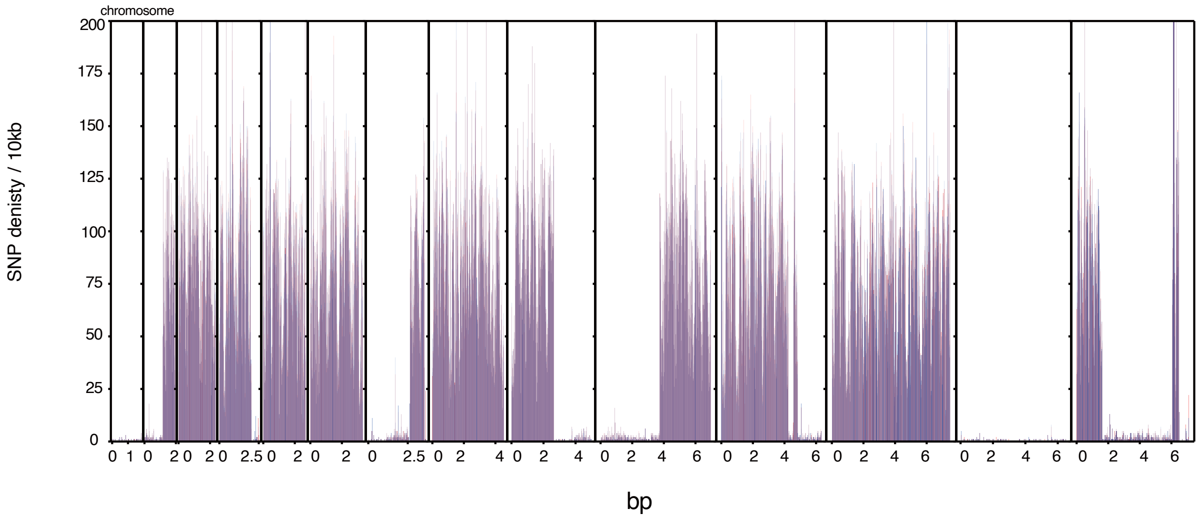

Figure S5. SNP density plot.

Pairwise comparison of SNPs for ME49 strains across 14 chromosomes. (a) red = TgCatJp5, blue = TgCatJpOk4; (b) red = VEG, blue = TgCatJpGi1/TaJ. Each vertical bar represents the number of SNPs within a 10 kbp window of the sequence.

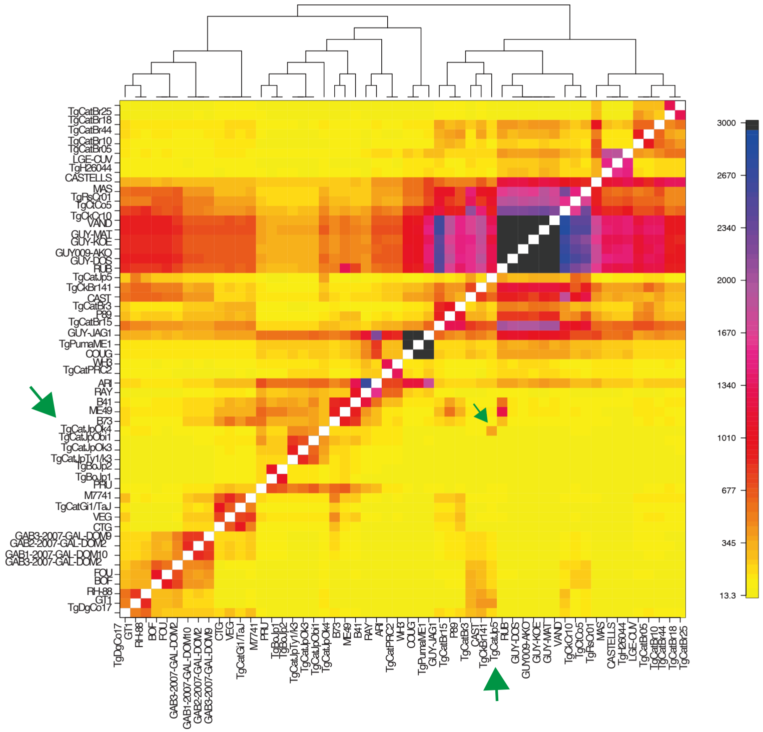

Figure S6. ChromoPainter co-ancestry matrix with population structure was assignment based on fineSTRUCTURE analysis.

The color of each cell of the matrix indicates the expected number of genetic materials copied from a donor genome (x axis) to a recipient genome (y axis). Green arrows indicate TgCatJpOk4, TgCatJp5, and the intersection of the two.

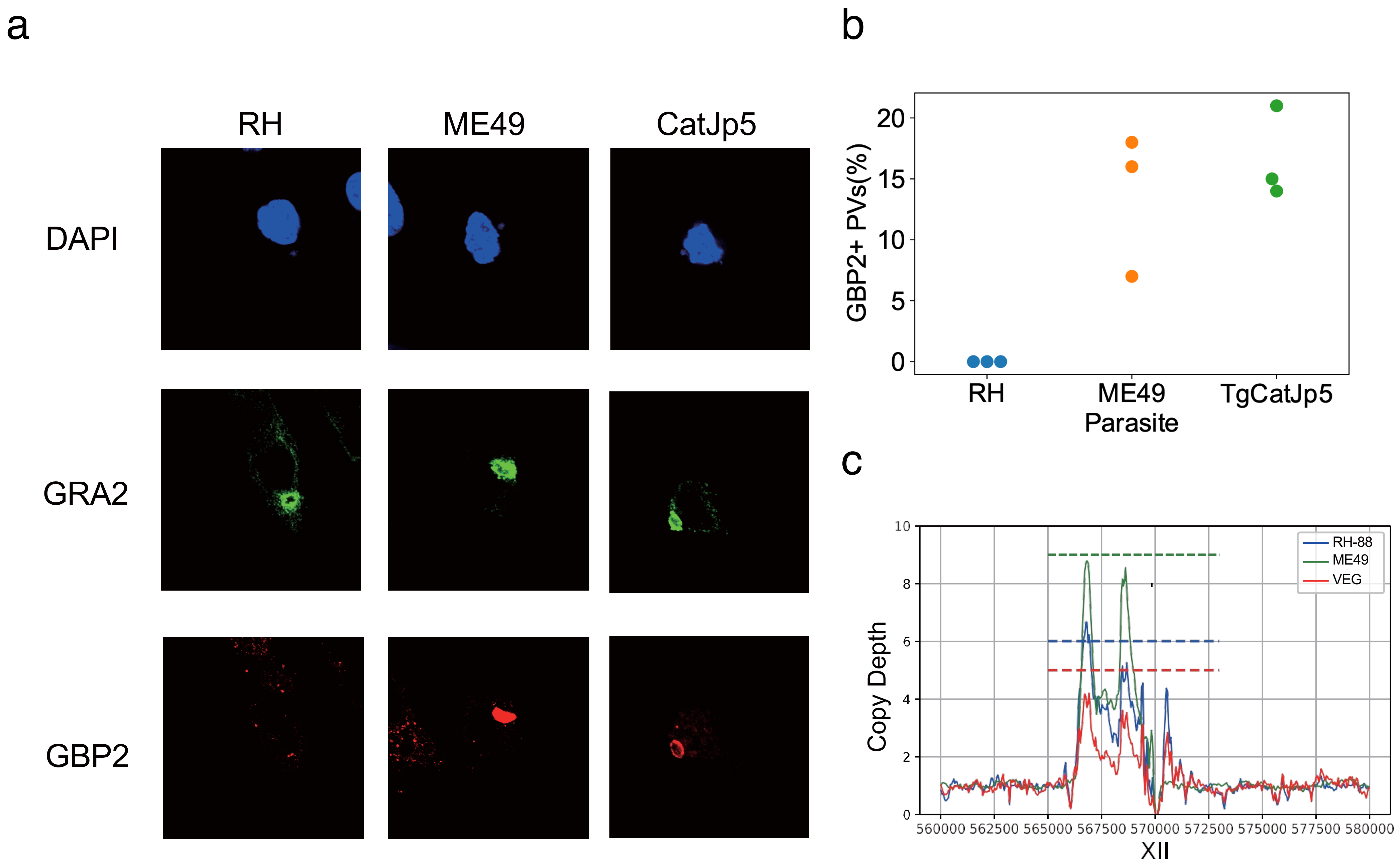

Figure S7. Accumulation of GBP2 on PV membranes.

(**a and b**) Accumulation of GBP2 into PV membrane was tested by infecting IFN-γ-stimulated mouse embryonic fibroblasts (MEFs) with the parasites HG1 (RH), HG2 (ME49), and TgCatJp5. Two hours later, cells were fixed, and immunofluorescence staining was performed using GRA2 (green), GBP2 (red), and DAPI (blue). The scale bar represents 10 µm.

(**c**) CNV analysis of ROP5 copies in RH-88, ME49, VEG. Copy number variation depth for *T. gondii* strains across the ROP5 locus on chromosome XII. Copy number estimates are based on the read depth per base pair normalized to 1X across the respective genome.

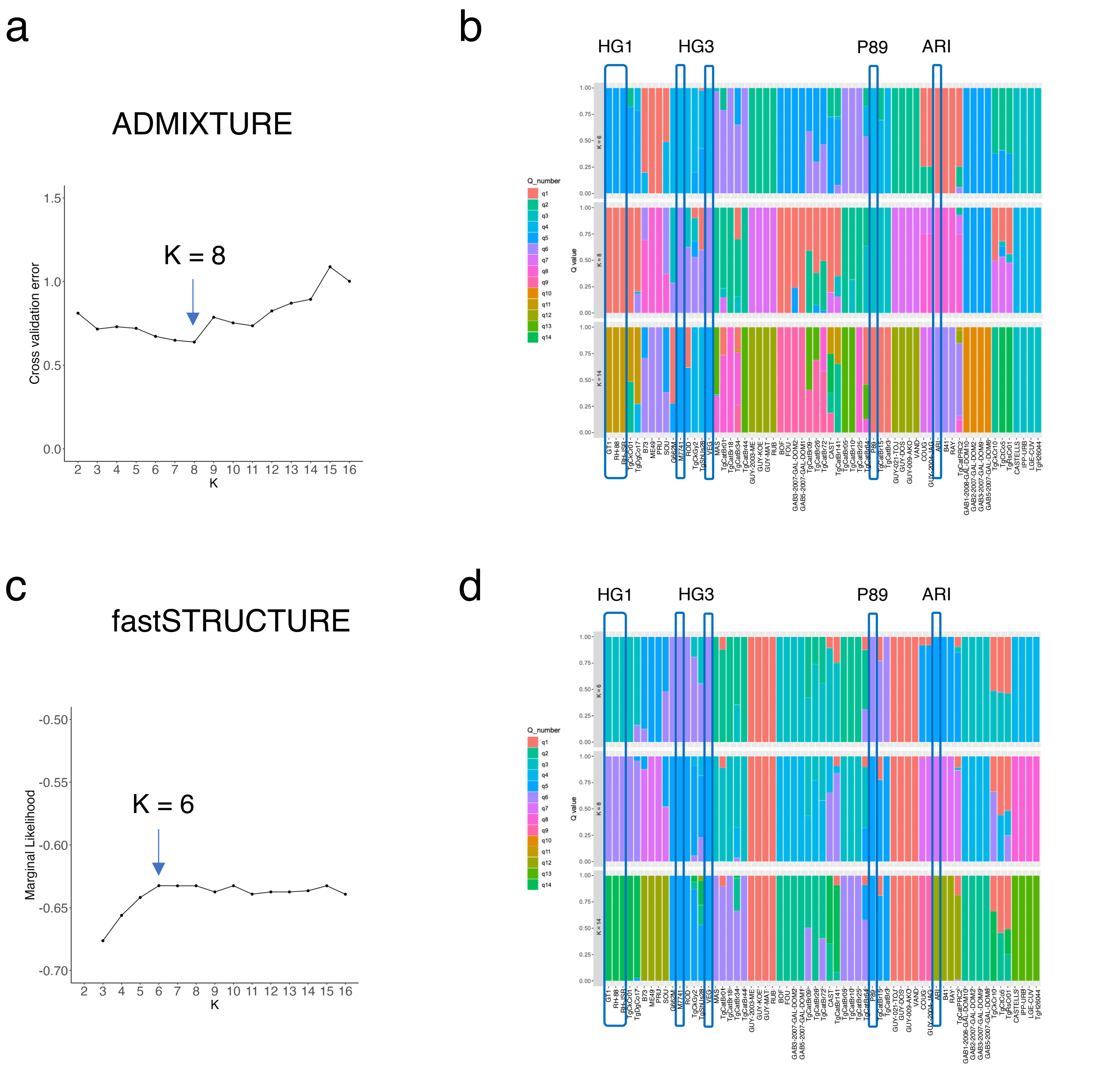

Figure S8. Benchmark analysis for evaluating ADMIXTURE and fastSTRUCTURE.

(a) Estimation of the number of ancestral populations (K) based on cross validation error.

(b) Clustering analysis of *T. gondii* was performed using ADMIXTURE. Results obtained at K = 6, 8, and 14 are shown. Each strain is represented by a vertical line composed of colored segments, in which each segment represents the proportion of an individuals' ancestry derived from one of the K ancestral populations. Strains are labeled on the bottom of the graph.

(c) Estimation of the number of ancestral populations (K) based on marginal likelihood.

(d) Clustering analysis of *T. gondii* was performed using fastSTRUCTURE.

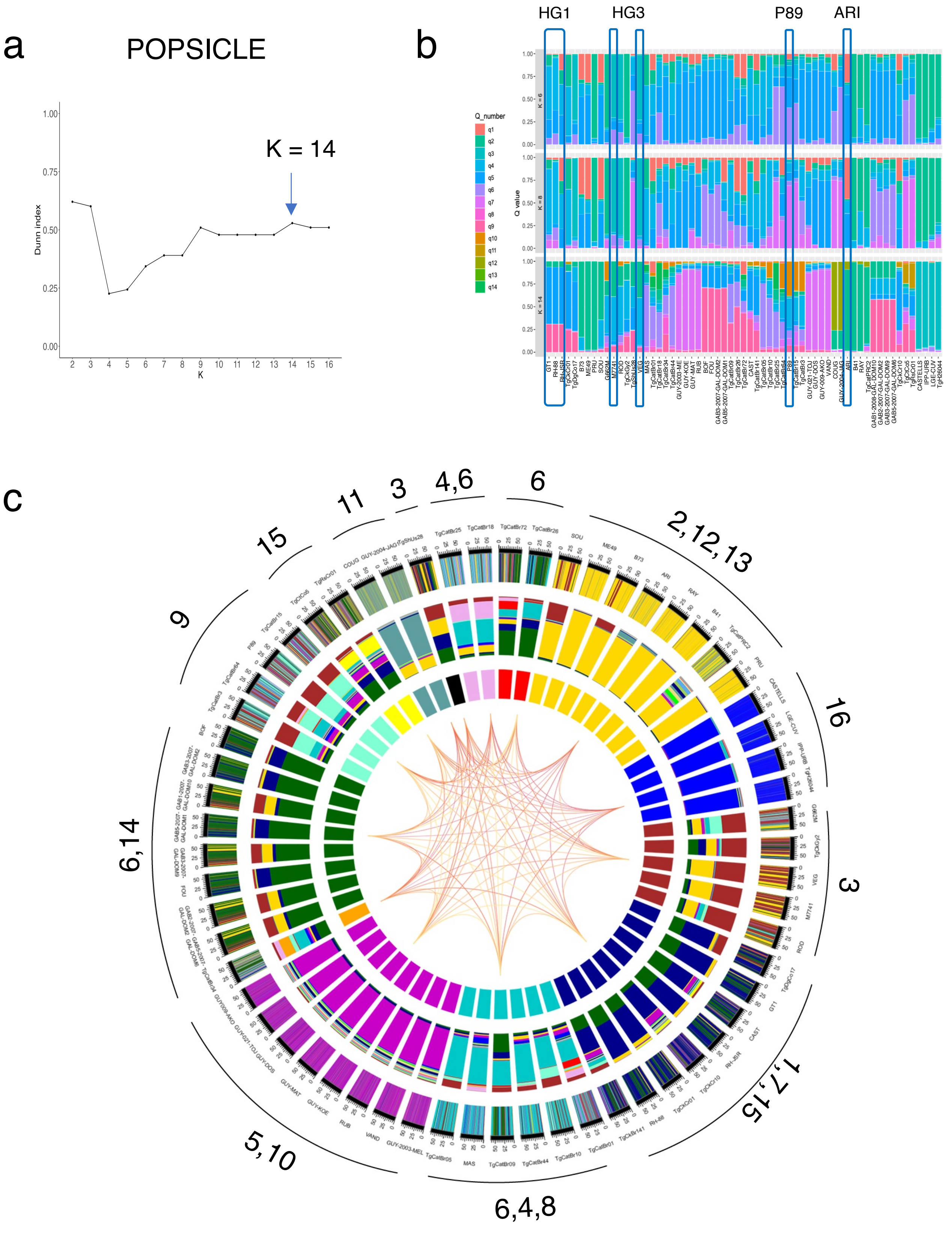

Figure S9. Benchmark analysis for evaluating POPSICLE.

(a) Estimation of the number of ancestral populations (K) based on Dunn index.

(b) Clustering analysis of *T. gondii* was performed using POPSICLE. Results obtained at K = 6, 8, and 14 are shown. Each strain is represented by a vertical line composed of colored segments, in which each segment represents the proportion of an individuals' ancestry derived from one of the K ancestral populations. Strains are labeled on the bottom of the graph.

(c) Population genetic structure and admixture clustering analysis of the *T. gondii* genomes obtained by POPSICLE using K = 14 different color hues in the innermost concentric circle of the Circos plot. The middle concentric circle shows the relative percentage of each genetic ancestry within each genome (represented by the color hues for K = 14). The outermost concentric circle shows the genome wide local admixture profile of each worm in 10 kb sliding windows. The haplotype plot with 61 strains identified lineages corresponding to existing genotypes (e.g. inner circle plot: dark blue (HG1), yellow (HG2), brown (HG3)).

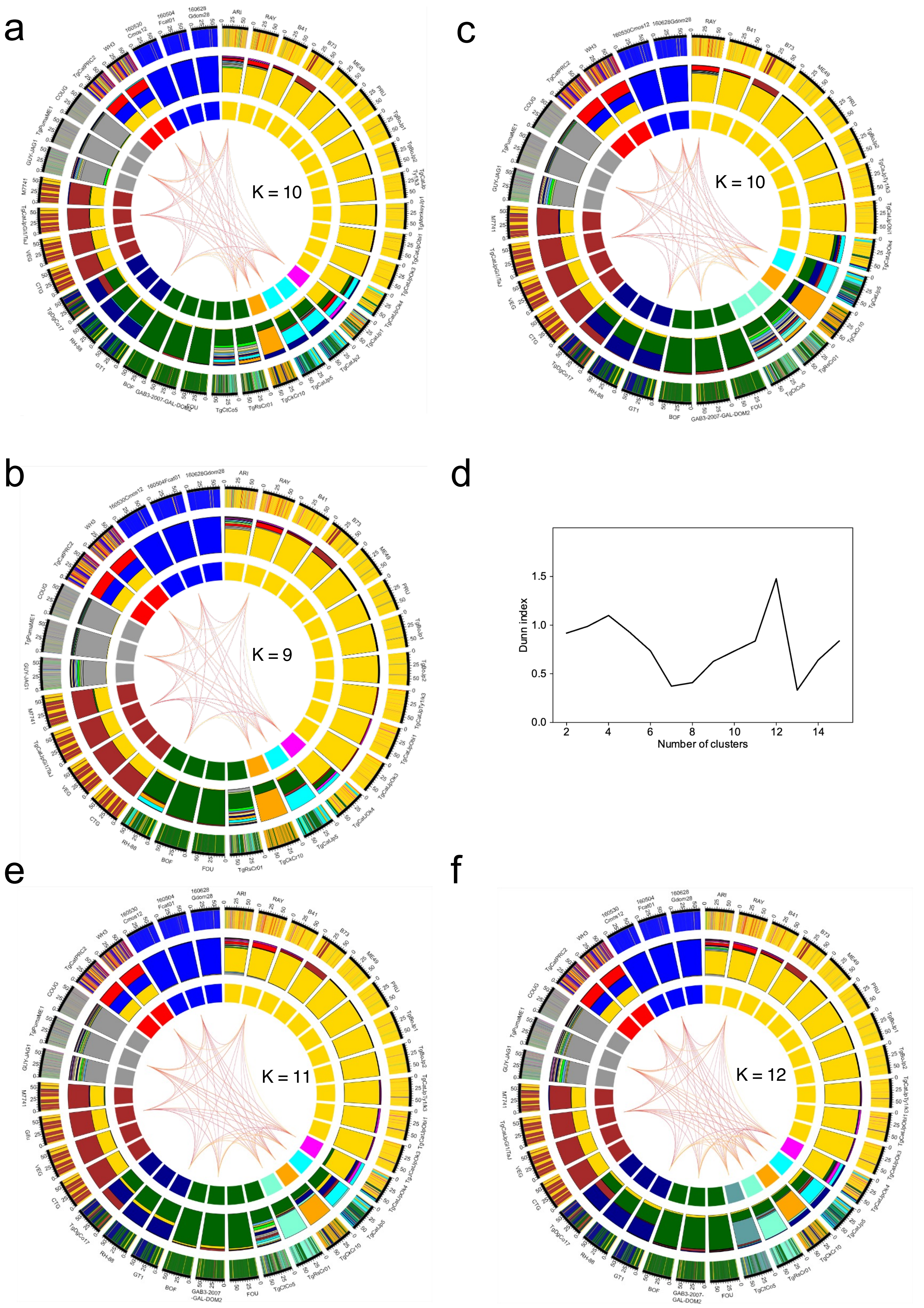
a
K = 10
c
K = 10
b
K = 9
d
Dunn index
Number of clusters
0.0
0.5
1.0
1.5
2
4
6
8
10
12
14
e
K = 11
f
K = 12

Figure S10. Additional POPSICLE plots.

(a) POPSICLE plot was made with the addition of TgCatJp1, TgCatJp2, and TgMonkeyJp1.

(b) POPSICLE plot was made excluding GAB3-2007-GAL-DOM2, GT1, TgCtCo5, and TgDgCo17.

(c) POPSICLE plot was made excluding 160504Fcat01, ARI, TgBoJp1, and TgCatJpOk3.

(d) Estimation of the number of ancestral populations (K) based on Dunn index.

(e) POPSICLE plot was made using k = 11.

(f) POPSICLE plot was made using k = 12.

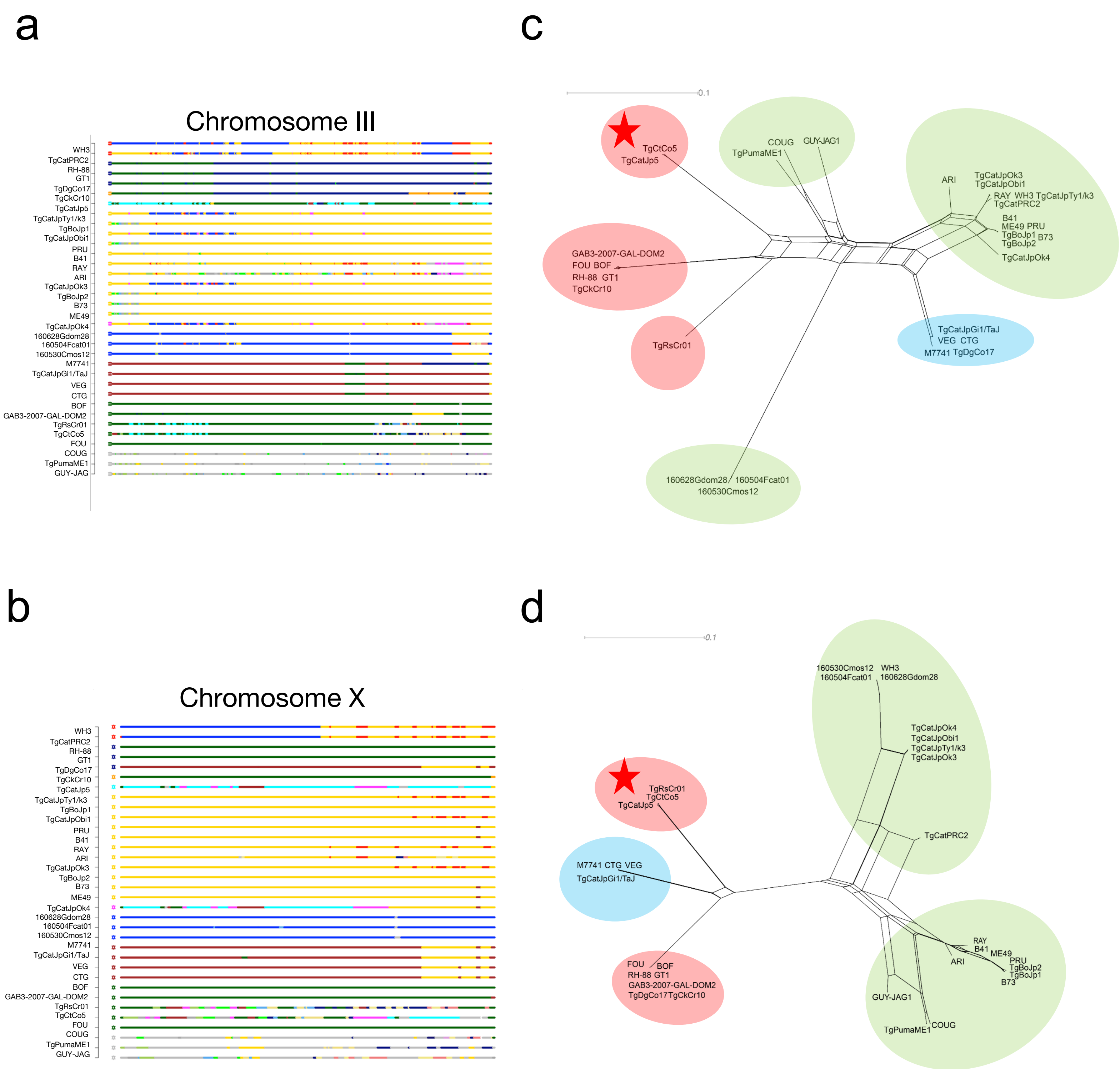

Figure S11. Phylogenetic analysis of ancestral haploblocks identified in the Japanese *T. gondii*.

(a) Chromosome painting of chromosome III.

(b) Chromosome painting of chromosome X.

(c) Neighbor-network based on the concatenated sequence of the portion designated as cyan in TgCatJp5 and TgCtCo5 in (a).

(d) Neighbor-network based on the concatenated sequence of the portion designated as cyan in TgCatJp5, TgRsCr01, and TgCtCo5 in (b). Color wheels indicate major clades of *T. gondii*.

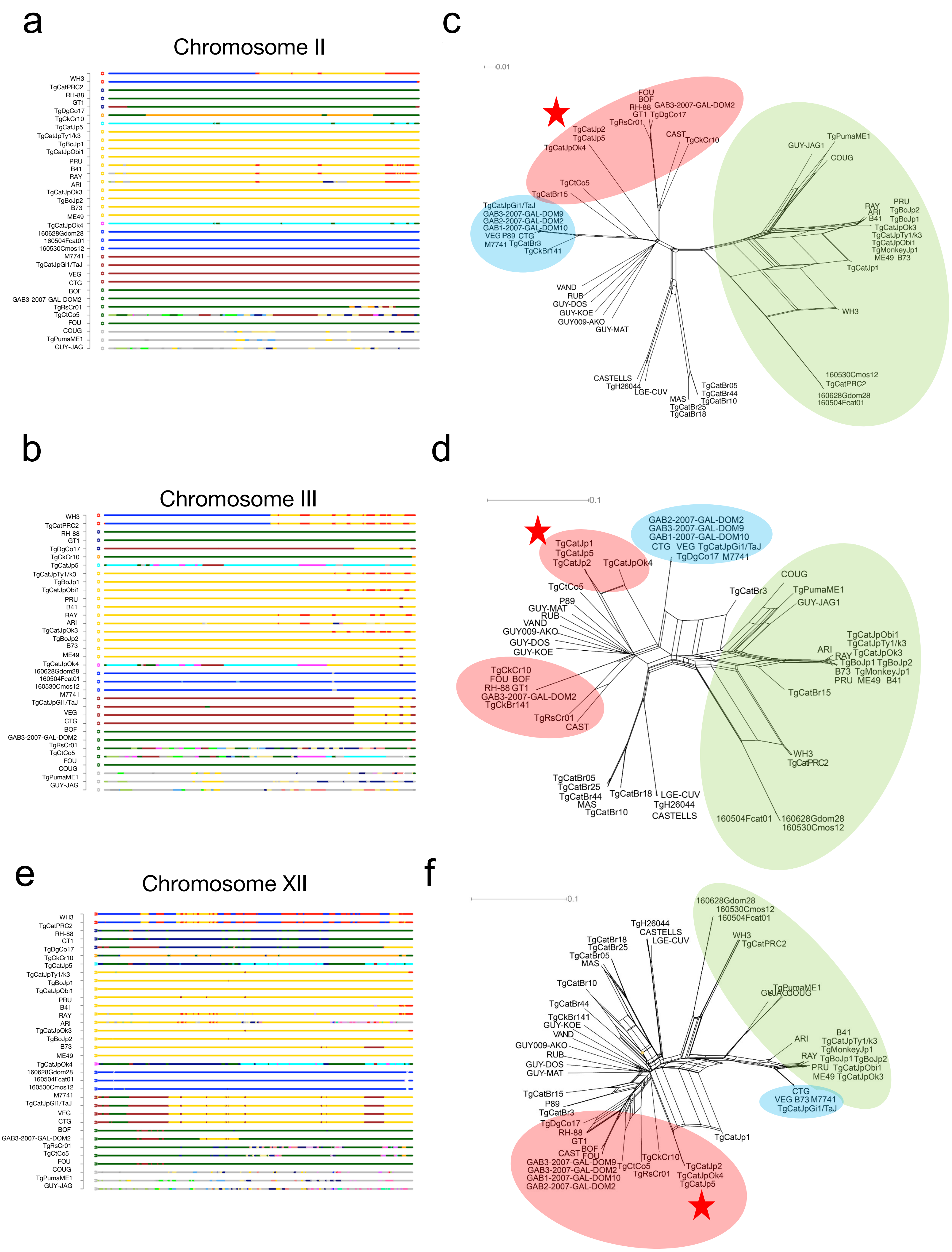
a
Chromosome II
c
b
Chromosome III
d
e
Chromosome XII
f

Figure S12. Phylogenetic analysis of ancestral haploblocks identified in the Japanese *T. gondii*.

(a) Chromosome painting of chromosome II.

(b) Chromosome painting of chromosome III.

(c) Chromosome painting of chromosome XII.

(d) Neighbor-network based on the concatenated sequence of the portion designated as cyan in TgCatJp5 and TgCatJpOk4 in (a).

(e) Neighbor-network based on the concatenated sequence of the portion designated as cyan in TgCatJp5, TgCatJpOk4 in (b).

(f) Neighbor-network based on the concatenated sequence of the portion designated as cyan in TgCatJp5, TgCatJpOk4 in (c). Color wheels indicate major clades of *T. gondii*.

a

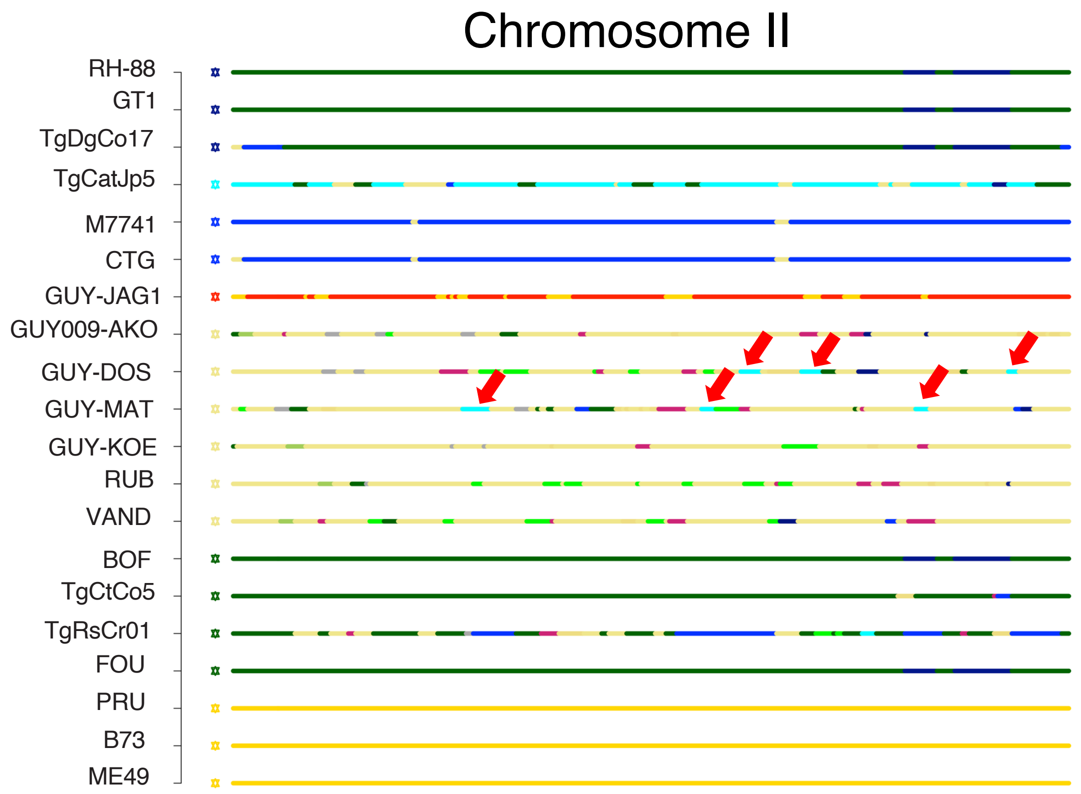

b

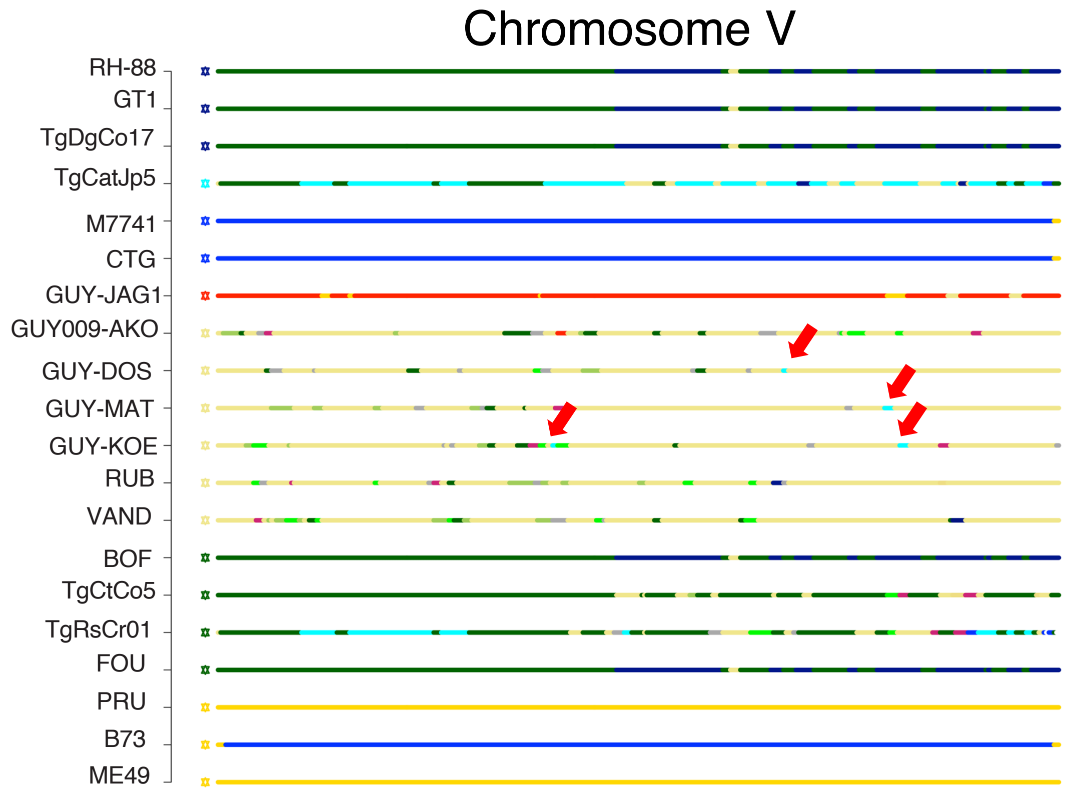

Figure S13. Chromosome painting based on POPSICLE plot with more South American strains.

(a) Chromosome painting of chromosome II.

(b) Chromosome painting of chromosome V. Red arrows indicate blocks designated as cyan in HG5 and HG10 strains.

Table S1. Summary of Target Enrichment Sequence.

Strain name	Alternative names	% of Toxoplasma DNA		Fold enrichment	Genome Covarage														% of SNPs that
		Before Enrichment	After enrichment	After/Before (x)	Average	chrIa	chrIb	chrII	chrIII	chrIV	chrV	chrVI	chrVIIa	chrVIII	chrIX	chrX	chrXI	chrXII	
TgCatJp1	Cat No.3	0.01549	49.2	3177	53.3	55.2	51.0	55.6	52.3	57.2	53.7	54.2	55.6	55.5	53.1	53.0	52.3	54.4	97%
TgCatJp2	Cat No.20	0.03111	26.5	852	32.8	35.5	32.4	33.6	33.8	37.1	34.2	32.8	33.9	32.9	32.4	34.3	33.1	32.4	92%
TgCatJp3	Cat No.21	0.00219	11.3	5169	12.5	13.5	12.1	12.9	12.8	14.4	13.4	12.3	12.9	12.5	12.0	13.2	12.6	12.2	74%
TgCatJp4	Cat No.30	0.00209	1.8	862	4.0	3.8	4.0	4.2	4.2	4.3	4.1	3.9	4.1	3.9	3.8	4.0	3.9	3.8	44%
TgCatJp6	Cat No.52	0.02731	3.2	117	7.7	8.2	7.5	8.3	8.0	9.3	8.7	7.7	8.0	7.8	7.4	8.4	7.9	7.6	60%
TgCatJp7	Cat No.55	0.00231	0.9	372	1.3	1.2	1.3	1.3	1.2	1.4	1.3	1.3	1.2	1.2	1.2	1.3	1.3	1.3	10%
TgMonkeyJp1	-	0.00096	52.9	54993	42.5	42.9	42.9	43.2	43.6	43.6	41.9	42.9	42.9	42.6	42.9	43.1	43.3	42.6	97%

Table S2. List of Toxoplasma isolates used in this study.

Haplogroup	Strain name	Alternative names	Geography	Year	Host	SRA Accession Numbers	Population analysis
HG1	RH-88		USA	1939	Human	SRX160126	○
	GT1		USA	1980	Goat	DRR513067	○
	TgDgCo17		Colombia	2006	Dog	SRX099787	○
HG2	ME49		USA	1965	Sheep	DRR513065	○
	PRU		France	1964	Human	SRX099792	○
	B73		USA	1994	Bear	SRX159844	○
HG3	VEG		USA	1988	Human	SRX156300	○
	M7741		USA	1976	Felis catus	SRX159890	○
	CTG		USA	1976	Animal	DRR513066	○
HG4	MAS		France	1991	Human	SRX057823 SRX038728 SRX038699	-
	TgCatBr44		Brazil	2008	Cat	SRX160141	-
	TgCatBr18		Brazil	2006	Cat	SRX099794	-
HG5	RUB	TgH00002, GUY-RUB	French Guiana	1991	Human	SRX055419 SRX055414 SRX099773 SRX055412	-
	GUY-KOE	TgH18002	French Guiana	2002	Human	SRX099796	-
	GUY-MAT	TgH18003	French Guiana	2002	Human	SRX099783	-
HG6	FOU	TgH20007	France	1992	Human	SRX046277 SRX038725 SRX046278	○
	BOF		Belgium	1993	Human	SRX099774	○
	GAB3-2007-GAL-DOM2	TgA105001	Gabon	2007	Chicken	SRX160123	○
HG7	CAST		USA	1988	Human	SRX099788	-
	TgCkBr141		Brazil	2006	Chicken	SRX160124	-
	-	-	-	-	-	-	-
HG8	TgCatBr5	TgCtBr5	Brazil	2006	Cat	SRX099804 SRX099805 SRX099795	-
	TgCatBr25	TgCtBr25	Brazil	2006	Cat	SRX160134	-
	TgCatBr10	TgCtBr10	Brazil	2006	Cat	SRX099791	-
HG9	P89	TgPgUs15	USA	1991	Pig	SRX038693 SRX055420 SRX038727	-
	TgCatBr3	TgCtBr15	Brazil	2003	Cat	SRX099779	-
	TgCatBr15	TgCtBr3	Brazil	2006	Cat	SRX160142	-
HG10	VAND	TgH00009, GUY-VAND	French Guiana	1997	Human	SRX055413 SRX038726 SRX055418 SRX055416	-
	GUY-DOS	TgH18001	French Guiana	2001	Human	SRX099782	-
	GUY009-AKO	TgH18009	French Guiana	2004	Human	SRX171132	-
HG11	COUG	TgCgCa1, TgCgCa01	Canada	1996	Cougar	SRX099803	○
	GUY-JAG1	TgA18001, GUY-JAG1	French Guiana	2004	Jaguar	SRX099776	○
	TgPumaME1		Mexico	2019	Puma	SRR366806	○
HG12	ARI		USA	1992	Human	SRR350724	○
	RAY		USA	1993	Human	SRX099793	○
	B41		USA	1994	Bear	SRX099774	○
HG13	TgCatPRC2		China	2007	Cat	SRX156168	○
	WH3		China	2011	Cat	DRR513081	○
	-	-	-	-	-		-
HG14	GAB2-2007-GAL-DOM2	TgA105004	Gabon	2007	Chicken	SRX156037 SRX155963 SRX155534	-
	GAB3-2007-GAL-DOM9	TgA105005	Gabon	2007	Chicken	SRX160125	-
	GAB1-2007-GAL-DOM10	TgA105003	Gabon	2007	Chicken	SRX159841	-
HG15	TgCtCo5		Colombia	2006	Cat	SRX156192 SRX156164 SRX156155 SRX154747	○
	TgCkCr10		Costa Rica	2006	Chicken	SRX099784	○
	TgRsCr1		Costa Rica	2006	Toucan	SRX160143	○
HG16	CASTELLS		Uruguay	1993	Sheep	SRX099789	-
	TgH21016	LGE-CUV	Unknown(Europ	2007	Human	SRX099775	-
	TgH26044		Unknown(Europ	2007	Human	SRX160050	-
Africa4(#20)	160504Fcat01	TgA117007	Senegal	2016	Cat	ERS13421665	○
	160530Cmos12	TgA117032	Senegal	2016	Duck	ERS13421666	○
	160628Gdom28	TgA117053	Senegal	2016	Chicken	ERS13421667	○
Japan	TgCatJpOk3		Okinawa	2013	Cat	DRX181171	○
	TgCatJpOk4		Okinawa	2013	Cat	DRX140735	○
	TgCatJp5	Cat No.49	Okinawa	2013	Cat	DRR513068	○
	TgCatJpTy1/k3		Tokyo	1986	Cat	DRR513069	○
	TgCatJpGi1/TaJ		Gifu	2013	Cat	DRR513070	○
	TgCatJpObi1		Hokkaido	2013	Cat	DRR513071	○
	TgBoJp1		Ehime	2023	Boar	DRR513072	○
	TgBoJp2		Ehime	2023	Boar	DRR513073	○
Enrichment sample	TgCatJp1	Cat No.3	Okinawa	2013	Cat	DRR513074	-
	TgCatJp2	Cat No.20	Okinawa	2013	Cat	DRR513075	-
	TgCatJp3	Cat No.21	Okinawa	2013	Cat	DRR513076	-
	TgCatJp4	Cat No.30	Okinawa	2013	Cat	DRR513077	-
	TgCatJp6	Cat No.52	Okinawa	2013	Cat	DRR513078	-
	TgCatJp7	Cat No.55	Okinawa	2013	Cat	DRR513079	-
	TgMonkeyJp1		Hokkaido	2011	Squirrel Monkey	DRR513080	-

Table S3. Number of alternative alleles for each lineage in the genomic regions assigned colors in Figure 6C.													
	**HG1**		**HG2**		**HG3**		**HG11**		**HG13**		**Africa4**		
Strain	GT1	RH-88	ME49	PRU	CTG	VEG	COUG	GUY-JAG1	TgCatPRC2	WH3	160504 Fcat1	160530 Cmos12	160628 Gdom28
Alt SNPS	125,878	125,207	404	2,324	69,182	68,621	86,090	80,446	82,500	83,169	128,689	128,198	132,245

Table S4. Percentage of ancestral clades of Africa 4blue, HG13red, and HG2yellow.				
	Strain	Africa 4^{blue}	HG13^{red}	HG2^{yellow}
**HG1**	TgDgCo17	0.0	0.0	7.3
	GT1	0.0	0.0	8.7
	RH-88	0.0	0.0	8.7
**HG2**	ME49	0.0	0.0	96.8
	B73	0.0	0.0	82.9
	PRU	0.0	0.1	97.1
**HG3**	CTG	0.0	0.0	39.4
	VEG	0.0	0.0	39.4
	Gifu	0.0	0.0	39.4
	M7741	0.0	0.0	38.6
**HG6**	FOU	0.0	0.0	4.0
	GAB3-2007-GAL-DOM2	0.0	0.0	6.5
	BOF	0.0	0.0	4.0
**HG11**	GUY-JAG1	0.3	0.0	3.7
	TgPumaME1	0.1	0.0	3.6
	COUG	0.1	0.0	3.9
**HG12**	ARI	0.1	6.9	66.2
	RAY	0.1	11.4	79.0
	B41	0.0	3.1	90.9
**HG13**	WH3	34.5	28.4	35.8
	TgCatPRC2	35.1	28.3	35.2
**HG15**	TgCtCo5	0.1	0.0	3.6
	TgRsCr01	0.1	0.0	2.5
	TgCkCr10	0.0	0.0	2.5
**Japanese Strains**	TgCatJpOk3	1.9	1.7	94.7
	TgCatJpTy1/k3	2.1	1.7	94.4
	TgCatObi1	2.0	1.2	92.4
	TgBoJp1	0.0	0.1	97.6
	TgBoJp2	0.0	0.1	97.5
	TgCatJpOk4	2.0	1.7	65.2
	TgCatJp5	0.0	2.4	9.2
