# Supplementary material for: Far-East Asian Toxoplasma isolates share ancestry with North and South/Central American recombinant lineages
Source: Nat Commun. 2024 May 22;15:4278. doi: 10.1038/s41467-024-47625-6 (PMC11111807; doi:10.1038/s41467-024-47625-6)
Supplement: Supplementary file 3 — Reporting Summary [file 41467_2024_47625_MOESM3_ESM.pdf]

nature portfolio

Corresponding author(s): Masahiro Yamamoto

Last updated by author(s): 2024/03/18

Reporting Summary

Nature Portfolio wishes to improve the reproducibility of the work that we publish. This form provides structure for consistency and transparency in reporting. For further information on Nature Portfolio policies, see our Editorial Policies and the Editorial Policy Checklist.

Statistics

For all statistical analyses, confirm that the following items are present in the figure legend, table legend, main text, or Methods section.

n/a	Confirmed	
☐	☒	The exact sample size (*n*) for each experimental group/condition, given as a discrete number and unit of measurement
☐	☒	A statement on whether measurements were taken from distinct samples or whether the same sample was measured repeatedly
☐	☒	The statistical test(s) used AND whether they are one- or two-sided<br>*Only common tests should be described solely by name; describe more complex techniques in the Methods section.*
☒	☐	A description of all covariates tested
☒	☐	A description of any assumptions or corrections, such as tests of normality and adjustment for multiple comparisons
☐	☒	A full description of the statistical parameters including central tendency (e.g. means) or other basic estimates (e.g. regression coefficient) AND variation (e.g. standard deviation) or associated estimates of uncertainty (e.g. confidence intervals)
☒	☐	For null hypothesis testing, the test statistic (e.g. *F*, *t*, *r*) with confidence intervals, effect sizes, degrees of freedom and *P* value noted<br>*Give P values as exact values whenever suitable.*
☒	☐	For Bayesian analysis, information on the choice of priors and Markov chain Monte Carlo settings
☒	☐	For hierarchical and complex designs, identification of the appropriate level for tests and full reporting of outcomes
☒	☐	Estimates of effect sizes (e.g. Cohen's *d*, Pearson's *r*), indicating how they were calculated

Our web collection on statistics for biologists contains articles on many of the points above.

Software and code

Policy information about availability of computer code

Data collection	No software was used to collect data in this study.
Data analysis	he following software or computer code was used for phylogenetic analysis<br>vcf2phyipl(M. Ortiz) SplitesTree4(v 4.18.2, DH. Hudson and D. Bryant) FigTree(v.14.4, A. Rambaut) MEGA(v11.0.13, K. TAMURA, G. STECHER,S. KUMAR) ModelTest-ng(v2.0.0,D. Dariiba) RAxML-ng(v1.1.0, M. Kozlov), ADMIXTURE(v.3.0, DH. Alexander) fastSTRUCTURE(v1.0, A. Raj) tinycov(v0.4.0, cmdoret)<br>・The following software was used for population analysis<br>fineSTRUCTURE(v2. https://people.maths.bris.ac.uk/~madjl/finestructure/finestructure_info.html)<br>POSICLE(https://POPSICLE-admixture.sourceforge.io), CIRCOS(http://circos.ca),<br>POPSILCE_ Admixtrue(https://sourceforge.net/projects/POPSICLE-admi xture/files/data/).<br>Python(v3.9.7) and R (v4.1.3) was used to generate heatmap, SNP density plot, and chromosome painting.<br>Custom scripts were available at DOI: 10.5281/zenodo.10785333, Foliium(v0.16.0, https://github.com/python-visualization/folium)

For manuscripts utilizing custom algorithms or software that are central to the research but not yet described in published literature, software must be made available to editors and reviewers. We strongly encourage code deposition in a community repository (e.g. GitHub). See the Nature Portfolio guidelines for submitting code & software for further information.

Data

Policy information about availability of data

All manuscripts must include a data availability statement. This statement should provide the following information, where applicable:

- Accession codes, unique identifiers, or web links for publicly available datasets
- A description of any restrictions on data availability
- For clinical datasets or third party data, please ensure that the statement adheres to our policy

DRR513065
[https://ddbj.nig.ac.jp/resource/sra-run/DRR513065]
DRR513066
[https://ddbj.nig.ac.jp/resource/sra-run/DRR513066]
DRR513067
[[https://ddbj.nig.ac.jp/resource/sra-run/DRR513067]
DRR513068
[https://ddbj.nig.ac.jp/resource/sra-run/DRR513068]
DRR513069
[https://ddbj.nig.ac.jp/resource/sra-run/DRR513069]
DRR513070
[https://ddbj.nig.ac.jp/resource/sra-run/DRR513070]
DRR513071
[https://ddbj.nig.ac.jp/resource/sra-run/DRR513071]
DRR513072
[https://ddbj.nig.ac.jp/resource/sra-run/DRR513072]
DRR513073
[https://ddbj.nig.ac.jp/resource/sra-run/DRR513073]
DRR513074
[https://ddbj.nig.ac.jp/resource/sra-run/DRR513074]
DRR513075
[https://ddbj.nig.ac.jp/resource/sra-run/DRR513075]
DRR513076
[https://ddbj.nig.ac.jp/resource/sra-run/DRR513076]
DRR513077
[https://ddbj.nig.ac.jp/resource/sra-run/DRR513077]
DRR513078
[https://ddbj.nig.ac.jp/resource/sra-run/DRR513078]
DRR513079
[https://ddbj.nig.ac.jp/resource/sra-run/DRR513079]
DRR513080
[https://ddbj.nig.ac.jp/resource/sra-run/DRR513080]
DRR513081
[https://ddbj.nig.ac.jp/resource/sra-run/DRR513081]
SRX160127
[https://www.ncbi.nlm.nih.gov/sra/?term=SRX160127]
SRX156300
[https://www.ncbi.nlm.nih.gov/sra/?term=SRX156300]
SRX099787
[https://www.ncbi.nlm.nih.gov/sra/?term=SRX099787]
SRX099792
[https://www.ncbi.nlm.nih.gov/sra/?term=SRX099792]
SRX159844
[https://www.ncbi.nlm.nih.gov/sra/?term=SRX159844]
SRX159890
[https://www.ncbi.nlm.nih.gov/sra/?term=SRX159890]
SRX057823
[https://www.ncbi.nlm.nih.gov/sra/?term=SRX057823]
SRX038728
[https://www.ncbi.nlm.nih.gov/sra/?term=SRX038728]
SRX038699
[https://www.ncbi.nlm.nih.gov/sra/?term=SRX038699]
TgCatBr44
[https://www.ncbi.nlm.nih.gov/sra/?term=SRX160141]
SRX099794
[https://www.ncbi.nlm.nih.gov/sra/?term=SRX099794]
SRX055419
[https://www.ncbi.nlm.nih.gov/sra/?term=SRX055419]

SRX055414,
[https://www.ncbi.nlm.nih.gov/sra/?term=SRX055414]
SRX099773
[https://www.ncbi.nlm.nih.gov/sra/?term=SRX099773]
SRX055412
[https://www.ncbi.nlm.nih.gov/sra/?term=SRX055412]
GUY-KOE
[https://www.ncbi.nlm.nih.gov/sra/?term=SRX099796]
SRX099783
[https://www.ncbi.nlm.nih.gov/sra/?term=SRX099783]
SRX099783
[https://www.ncbi.nlm.nih.gov/sra/?term=SRX099783]
SRX038725
[https://www.ncbi.nlm.nih.gov/sra/?term=SRX038725]
SRX046278
[https://www.ncbi.nlm.nih.gov/sra/?term=SRX046278]
SRX099774
[https://www.ncbi.nlm.nih.gov/sra/?term=SRX099774]
SRX160123
[https://www.ncbi.nlm.nih.gov/sra/?term=SRX160123]
SRX099788
[https://www.ncbi.nlm.nih.gov/sra/?term=SRX099788]
SRX160124
[https://www.ncbi.nlm.nih.gov/sra/?term=SRX160124]
SRX099804
[https://www.ncbi.nlm.nih.gov/sra/?term=SRX099804]
SRX099805
[https://www.ncbi.nlm.nih.gov/sra/?term=SRX099805]
SRX099795
[https://www.ncbi.nlm.nih.gov/sra/?term=SRX099795]
SRX160134
[https://www.ncbi.nlm.nih.gov/sra/?term=SRX160134]
SRX099791
[https://www.ncbi.nlm.nih.gov/sra/?term=SRX099791]
SRX038693
[https://www.ncbi.nlm.nih.gov/sra/?term=SRX038693]
SRX055420
[https://www.ncbi.nlm.nih.gov/sra/?term=SRX055420]
SRX038727
[https://www.ncbi.nlm.nih.gov/sra/?term=SRX038727]
SRX099779
[https://www.ncbi.nlm.nih.gov/sra/?term=SRX099779]
SRX160142
[https://www.ncbi.nlm.nih.gov/sra/?term=SRX160142]
SRX055413
[https://www.ncbi.nlm.nih.gov/sra/?term=SRX055413]
SRX038726
https://www.ncbi.nlm.nih.gov/sra/?term=SRX038726]
SRX055418
[https://www.ncbi.nlm.nih.gov/sra/?term=SRX055418]
SRX055416
[https://www.ncbi.nlm.nih.gov/sra/?term=SRX055416]
SRX099782
[https://www.ncbi.nlm.nih.gov/sra/?term=SRX099782]
SRX171132
[https://www.ncbi.nlm.nih.gov/sra/?term=SRX171132]
SRX099803
[https://www.ncbi.nlm.nih.gov/sra/?term=SRX099803]
SRX099776
[https://www.ncbi.nlm.nih.gov/sra/?term=SRX099776]
SRR366806
[https://www.ncbi.nlm.nih.gov/sra/?term=SRR366806]
SRR350724
[https://www.ncbi.nlm.nih.gov/sra/?term=SRR350724]
SRX099793
[https://www.ncbi.nlm.nih.gov/sra/?term=SRX099793]
SRX099774
[https://www.ncbi.nlm.nih.gov/sra/?term=SRX099774]

SRX156168
[https://www.ncbi.nlm.nih.gov/sra/?term=SRX156168]
SRX156037
[https://www.ncbi.nlm.nih.gov/sra/?term=SRX156037]
SRX155963
[https://www.ncbi.nlm.nih.gov/sra/?term=SRX155963]
SRX155534
[https://www.ncbi.nlm.nih.gov/sra/?term=SRX155534]
SRX160125
[https://www.ncbi.nlm.nih.gov/sra/?term=SRX160125]
SRX159841
[https://www.ncbi.nlm.nih.gov/sra/?term=SRX159841]
SRX156192
[https://www.ncbi.nlm.nih.gov/sra/?term=SRX156192]
SRX156164
[https://www.ncbi.nlm.nih.gov/sra/?term=SRX156164]
SRX156155
[https://www.ncbi.nlm.nih.gov/sra/?term=SRX156155]
SRX154747
[https://www.ncbi.nlm.nih.gov/sra/?term=SRX154747]
SRX099784
[https://www.ncbi.nlm.nih.gov/sra/?term=SRX099784]
SRX160143
[https://www.ncbi.nlm.nih.gov/sra/?term=SRX160143]
SRX099789
[https://www.ncbi.nlm.nih.gov/sra/?term=SRX099789]
SRX099775
[https://www.ncbi.nlm.nih.gov/sra/?term=SRX099775]
SRX160050
[https://www.ncbi.nlm.nih.gov/sra/?term=SRX160050]
ERS13421665
[https://www.ncbi.nlm.nih.gov/sra/?term=ERS13421665]
ERS13421666
[https://www.ncbi.nlm.nih.gov/sra/?term=ERS13421666]
ERS13421667
[https://www.ncbi.nlm.nih.gov/sra/?term=ERS13421667]
Nucleus genome referenced ToxoDB-57-T.gondiiME49
[https://toxodb.org/common/downloads/release-57/TgondiiME49/fasta/data/ToxoDB-57_TgondiiME49_Genome.fasta]
Apicoplast genome referenced ToxoDB-61-T.gondiiRH88
[https://toxodb.org/common/downloads/release-61/TgondiiRH88/fasta/data/ToxoDB-61_TgondiiRH88_Genome.fasta]
The raw data for all data presented in graphs in this study are provided in the Source Data file.

Research involving human participants, their data, or biological material

Policy information about studies with human participants or human data. See also policy information about sex, gender (identity/presentation), and sexual orientation and race, ethnicity and racism.

Reporting on sex and gender	Not applicable
Reporting on race, ethnicity, or other socially relevant groupings	Not applicable
Population characteristics	Not applicable
Recruitment	Not applicable
Ethics oversight	Not applicable

Note that full information on the approval of the study protocol must also be provided in the manuscript.

Field-specific reporting

Please select the one below that is the best fit for your research. If you are not sure, read the appropriate sections before making your selection.

☒ Life sciences ☐ Behavioural & social sciences ☐ Ecological, evolutionary & environmental sciences

For a reference copy of the document with all sections, see nature.com/documents/nr-reporting-summary-flat.pdf

Life sciences study design

All studies must disclose on these points even when the disclosure is negative.

Sample size	The number of animals was determined based on the number of animals used in previously published work.
Data exclusions	No data were excluded from the results obtained in this study.
Replication	Each experiment was performed with at least 3 biological replicates.<br>All replication attempts yielded similar results and were reliably reproduced.
Randomization	All samples and animals were randomly allocated into experimental groups.
Blinding	No blinded testing was performed because the same investigator set up the experiments, collected the samples, and analyzed the data. However, the samples and data were collected under identical conditions.

Reporting for specific materials, systems and methods

We require information from authors about some types of materials, experimental systems and methods used in many studies. Here, indicate whether each material, system or method listed is relevant to your study. If you are not sure if a list item applies to your research, read the appropriate section before selecting a response.

Materials & experimental systems

n/a	Involved in the study	
☐	☒	Antibodies
☐	☒	Eukaryotic cell lines
☒	☐	Palaeontology and archaeology
☐	☒	Animals and other organisms
☒	☐	Clinical data
☒	☐	Dual use research of concern
☒	☐	Plants

Methods

n/a	Involved in the study	
☒	☐	ChIP-seq
☒	☐	Flow cytometry
☒	☐	MRI-based neuroimaging

Antibodies

Antibodies used	1 Rabbit polyclonal anti-GRA7 (1:100) was provided by Dr. C. Boothroyd (Stanford University)<br>2 Mouse monoclonal anti-GRA2 (1:100) was provided by Dr. Soldati-Favre(University of Geneva)<br>3 Goat polyclonal anti-Irgb6(10D7) (1:100) (sc11079, Santa Cruz Biotechnology, USA)<br>4 Rabbit polyclonal anti-GBP2 (1:100) (H00008878, Proteintech, USA)<br>5 Secondary antibodies (1:2500) for immunostaining; Alexa Fluor 488 (anti-Mouse, Cat#20014, Biotium, USA) , Alexa Fluor 488 (anti-Rabbit,Cat#20015, Biotium, USA), Alexa Fluor 594 (anti-Goat, Cat#A11058, Invitrogen, USA), and Alexa Fluor 594 (anti-Rabbit, Cat#20152, Biotium, USA)
Validation	1- 4: Hashizaki et al. 2023 (https://journals.asm.org/doi/full/10.1128/mbio.03256-22?rfr_dat=cr_pub++0pubmed&url_ver=Z39.88-2003&rfr_id=ori%3Arid%3Acrossref.org)<br>5 https://biotium.com/product/donkey-anti-mouse-igg-hl-highly-cross-adsorbed/<br>https://biotium.com/product/donkey-anti-rabbit-igg-hl-highly-cross-adsorbed/<br>https://www.thermofisher.com/antibody/product/Donkey-anti-Goat-IgG-H-L-Cross-Adsorbed-Secondary-Antibody-Polyclonal/A-11058<br>https://biotium.com/product/donkey-anti-rabbit-igg-hl-highly-cross-adsorbed/

Eukaryotic cell lines

Policy information about cell lines and Sex and Gender in Research

Cell line source(s)	ATCC, a primary cells of murine (C57BL/6, female), RIKEN BRC CELL BANK
Authentication	Cell line authentication was initially performed by ATCC or RIKEN BRC CELL BANK. Further authentication was performed by microscopy, as two cell lines used in this study (HFF, Vero) have quite distinct morphology.<br>MEF cell was isolated from a mouse in a previous study (Hashizaki et al. 2023).
Mycoplasma contamination	The cell lines were not tested for mycoplasma contamination.
Commonly misidentified lines (See ICLAC register)	No commonly misidentified cell lines were used in this study.

Animals and other research organisms

Policy information about studies involving animals; ARRIVE guidelines recommended for reporting animal research, and Sex and Gender in Research

Laboratory animals	Mouse (ICR and C57BL/6, and Ifngr1-/-), 8 to 12-weeks old female mice.
Wild animals	2 wild boars, The animals were captured in an enclosure trap, a box trap, or a snare trap for the purpose of agricultural animal damage control. The animals euthanized by bloodletting after syncope with gunshot or electric fainting as soon as the capture was confirmed. The carcasses were dismembered at near facility. The samples were collected at that time and stored on refrigerated or frozen. Some of the animals collected were used as food for private consumption.
Reporting on sex	Female mice were used for all experiment in this study<br><br>Wild boars were both male, 1 year-old.
Field-collected samples	No field-collected samples were used in the study.
Ethics oversight	All animal experiments were performed with the approval of the Animal Research Committee of the Research Institute for Microbial Diseases, Osaka University (Permission number: R03-20-0).

Note that full information on the approval of the study protocol must also be provided in the manuscript.

Plants

Seed stocks	Not applicable
Novel plant genotypes	Not applicable
Authentication	Not applicable
